# Multiplex immunohistochemistry defines two cholesterol metabolism patterns predicting immunotherapeutic outcomes in gastric cancer

**DOI:** 10.1186/s12967-023-04758-4

**Published:** 2023-12-07

**Authors:** Wei Tang, Guanghua Li, Qi Lin, Zhenzhen Zhu, Zhao Wang, Zhixiong Wang

**Affiliations:** 1 Department of Gastrointestinal Surgery, First Affiliated Hospital of Sun Yat-sen University, Zhongshan 2nd Street, No. 58, Guangzhou, 510080 Guangdong China; 2 Stroke Center, Panyu Central Hospital, Fuyu East Street No. 8, Guangzhou, 511400 Guangdong China

**Keywords:** Gastric cancer, Cholesterol metabolism, Metabolic subtypes, Immunotherapy, Prognostic signature, Biomarkers

## Abstract

**Background:**

The role of cholesterol metabolism in gastric cancer (GC) and its implications for tumor characteristics and immunotherapy response remain poorly understood. In this study, our aim was to investigate this role, identify associated metabolic subtypes, and assess their clinical implications in GC.

**Methods:**

We conducted a comprehensive analysis of cholesterol metabolism genes (CMGs) using transcriptomic data from TCGA and GEO. Based on 23 representative CMGs, we classified GC into metabolic subtypes. We evaluated clinical features and immune cell infiltration between these subtypes. Additionally, we identified a CMG signature and assessed its clinical relevance in GC. We retrospectively enrolled thirty-five GC patients receiving chemotherapy plus a PD-1 inhibitor to assess the CMG signature using multiplex immunohistochemistry.

**Results:**

Our analysis revealed two cholesterol metabolism subtypes in GC: Cholesterol Metabolism Type 1 (CMT1) and Cholesterol Metabolism Type 2 (CMT2). These subtypes exhibited distinct patterns: CMT1 indicated heightened cholesterol biosynthesis, while CMT2 showed abnormal cholesterol transport. CMT2 was associated with unfavorable clinical features, enriched malignant pathways, and a pro-tumor immune microenvironment. Furthermore, we developed a five-CMG prognostic signature (ABCA1, NR1H3, TSPO, NCEH1, and HMGCR) that effectively predicted the prognosis of patients with GC and their response to chemotherapy plus a PD-1 inhibitor. This signature was validated in a clinical cohort using multiplex immunohistochemistry.

**Conclusion:**

Our results highlight the effectiveness of cholesterol metabolism patterns as biomarkers for predicting the prognosis and immunotherapy response in GC. The expression of cholesterol metabolism genes and the assessment of cholesterol metabolism patterns have the potential to predict the outcome of immunotherapy and guide treatment strategies.

**Supplementary Information:**

The online version contains supplementary material available at 10.1186/s12967-023-04758-4.

## Introduction

Cholesterol metabolism reprogramming has been implicated in numerous diseases, including cancer, and its association with tumor cell proliferation, migration, and invasion has been demonstrated [1]. In cancer, cholesterol plays a variety of roles. First, as one of the metabolic materials, the excess cholesterol in tumor cells meets the energy and biosynthesis requirements for rapid proliferation [2, 3]. In addition, cholesterol can accumulate in membrane microstructures known as lipid rafts, which provide a dynamic signaling platform rich in growth factor receptors and adhesion molecules involved in regulating cell proliferation, migration, and chemotherapy response [4–7]. Moreover, numerous studies have found that cholesterol metabolism can shape the tumor microenvironment (TME) by affecting the phenotype and function of stromal cells, particularly tumor-infiltrating immune cells [8]. For instance, a study by Ma et al. found that cholesterol released by tumor cells increased endoplasmic reticulum (ER) stress in CD8+T cells in the TME, resulting in upregulated immune checkpoint expression and induction of CD8+T-cell exhaustion [9]. In addition, for intracellular cholesterol metabolism in immune cells, Yang et al. discovered that inhibition of acetyl-CoA acetyltransferase 1 (ACAT1) can enhance the antitumor response of CD8+T cells [10]. These findings reflect a significant relationship between cholesterol metabolism and tumor immunity.

Cholesterol metabolism is a complex process involving biosynthesis, uptake, storage, esterification, and efflux of cholesterol [11]. Dysregulation and reprogramming in any of these steps can be found in tumors [12]. Both cholesterol synthesis and transport pathways are directly related to cholesterol levels in tumor cells and have therefore been extensively studied. However, the main mechanism of abnormal cholesterol metabolism can vary depending on the type of tumor. For example, squalene epoxidase (SQLE), one of the rate-limiting enzymes in cholesterol synthesis, has been linked to tumor development and has been investigated as a potential therapeutic target [13–16]. In various cancer types, Niemann–Pick type C-1 (NPC1), a lysosomal cholesterol transporter, has also been extensively studied. By blocking its function, itraconazole prevents the release of cholesterol from lysosomes, thereby reducing tumor growth and angiogenesis [17–20]. Meanwhile, the association between cholesterol metabolism and gastric cancer (GC) has also been reported. The expression of sterol *O*-acyltransferase 1 (SOAT1), a protein associated with cholesterol synthesis, has been found to promote lipid synthesis and lymph node metastasis in GC [21]. Studies have also demonstrated the upregulation of various cholesterol metabolic factors, such as HMG-CoA reductase (HMGCR) and apolipoprotein E (ApoE), in GC, which correlates with poor prognosis [22, 23]. These findings indicate the presence of abnormal cholesterol metabolism in GC. However, our understanding of the abnormal metabolic pattern of cholesterol in GC is still limited, and to our knowledge, few reports have been published.

At present, there are more and more advanced disease diagnosis and treatment technologies, such as the application of green nanomaterials [24]. The study of human metabolism and disease is also deepening [3, 25]. Preclinical research based on bioinformatics and laboratory validation enhances our understanding of cancer, providing a nuanced perspective that complements existing advanced technologies. In this study, we collected a gene set consisting of genes involved in the synthesis, transport, and related regulatory factors of cholesterol metabolism. By analyzing these cholesterol metabolism genes (CMGs), we identified two distinct patterns of cholesterol metabolism in GC: cholesterol metabolism type 1 (CMT1) and cholesterol metabolism type 2 (CMT2). CMT1 was characterized by active cholesterol synthesis, while CMT2 was characterized by abnormal cholesterol transport. Based on public databases, we analyzed the differences in several features between the two subtypes of GC using bioinformatics analysis. Additionally, the results of these analyses were validated by multiplex immunohistochemistry in our clinical cohort. Our findings provide novel insights into the patterns of cholesterol metabolism in GC and expand our understanding of the heterogeneity of GC, which could lead to the identification of potential therapeutic targets for this disease.

## Materials and methods

### Dataset collection and preprocessing

Public data of patients with GC were collected from The Cancer Genome Atlas (TCGA) database (https://cancergenome.nih.gov/) and NCBI Gene Expression Omnibus (GEO) database (https://www.ncbi.nlm.nih.gov/geo/). From the TCGA database, RNA sequencing data of 407 patients with GC (selected condition: Program-TCGA, Disease type-Adenomas and Adenocarcinomas) were downloaded. The data were then converted to transcripts per kilobase million (TPM) format. Matched clinical information, somatic mutation data, and Copy number variation (CNV) data files were also collected. From the GEO database, the RNA sequencing data and the matched clinical information of 300 patients with advanced GC (the Asian Cancer Research Group (ACRG) cohort, GSE62254) were collected [26]. We also downloaded GSE84437 as a validation set. The above data was obtained on October 10, 2022.

In addition, cholesterol metabolism genes (CMGs) were obtained from the Molecular Signature Database (MSigDB, https://www.gsea-msigdb.org) and Kyoto Encyclopedia of Genes and Genomes (KEGG, https://www.kegg.jp/entry/hsa04979). A total of 23 CMGs (including ABCA1, ABCG1, CETP, FDPS, HMGCR, HMGCS1, LCAT, LDLR, LSS, NCEH1, NPC1, NPC2, NR1H2, NR1H3, PCSK9, SC5D, SCARB1, SOAT1, SQLE, SREBF1, SREBF2 and TSPO) were chosen to compose the gene set (shown in Additional file 1: Table S1).

### Cluster analysis

Using the 23 CMGs, we conducted cluster analysis with the R package 'ConsensuClusterPlus' [27] to identify distinct cholesterol metabolism patterns and divide patients from the ACRG cohort into different groups. We determined the optimal number of clusters by selecting the k value that minimized the within-cluster sum of squares and then confirmed the stability of the classification by performing 1000 repetitions. Survival analysis was performed using Kaplan‒Meier curves and log-rank tests to compare differences in survival between subgroups. We also compared the distribution of clinical features (including age, sex, TNM stage, Lauren type, and molecular subtype) between subgroups. We repeated this analysis on the TCGA-STAD cohort to validate the repeatability of the clustering.

The differentially expressed genes (DEGs) between subgroups were screened out using the limma package in R with criteria of |log FC|> 0.1 and an adjusted P value < 0.05. Then, the molecular functions of DEGs were investigated using Gene Ontology (GO) analysis and Gene Set Enrichment Analysis (GSEA). Furthermore, we employed gene set variation analysis (GSVA) [28] using the KEGG gene set (c2.cp.kegg. v2022.1, MSigDB) to compare differences in biological functional enrichment between subgroups (adjusted P value < 0.05).

### Estimation of immune cell infiltration, immune checkpoints, and immunotherapy response

We estimated immune cell infiltration by employing several algorithms in R: each sample's ESTIMATE score, Immune score, Stromal score and tumor purity were determined using the R package 'ESTIMATE' [29]. The single-sample gene-set enrichment analysis (ssGSEA) algorithm was utilized to quantify the relative abundance of each immune cell infiltration [30], and the CIBERSORT [31] tool was employed to determine the proportions of 23 different immune cell types.

Immune checkpoints (CTLA4 (Cytotoxic T-Lymphocyte Associated Antigen 4), PD-1 (Programmed Cell Death Protein 1), PD-L1 (Programmed Cell Death Protein 1), LAG3 (Lymphocyte Activation Gene 3), TIM3 (T Cell Immunoglobulin Mucin Receptor 3) and TIGIT (T Cell Immunoreceptor With Ig And ITIM Domains Protein)) and were studied to determine their relationships with cholesterol metabolism types. To predict patients' response to immune checkpoint blockade therapy, the tumor mutation burden (TMB) the of the TCGA-STAD cohort was calculated, and the Tumor Immune Dysfunction and Exclusion (TIDE) score of was generated using the TIDE algorithm [32] (http://tide.dfci.harvard.edu/).

### Identification of prognostic CMGs

The potential prognostic CMGs with statistical significance were screened out by univariate Cox analysis (P < 0.05). Then, the Lasso Cox regression model was applied to obtain the regression coefficients for these potential prognostic genes [33, 34]. Subsequently, we created a risk model using the regression coefficients from the multivariate Cox regression analysis. The formula for the risk model was established as follows: *Risk score* = *expression(gene[1])* × *coefficient(gene[1])* + *expression(gene[2])* × *coefficient(gene[2])* + *…* + *expression(gene[n])* × *coefficient(gene[n])*. Kaplan‒Meier survival analysis and multivariate Cox analysis were performed to evaluate the prognostic value of the risk model.

### Sample acquisition for clinical validation

To validate our results, we retrospectively collected formalin-fixed paraffin-embedded (FFPE) tissues from 35 patients with GC who were diagnosed between January 1st, 2019, and May 1st, 2023, in the First Affiliated Hospital of Sun Yat-sen University. The inclusion criteria were as follows: (i) an Eastern Cooperative Oncology Group performance status of 0–1; (ii) histologically proven, unresectable, locally advanced, or metastatic GC; (iii) negative HER-2 expression; (iv) received at least three cycles of chemotherapy combined with an anti-PD-1 ICI antibody (Pembrolizumab, Nivolumab, Camrelizumab, Sintilimab or Tislelizumab); (v) available FFPE tumor tissue acquired from endoscopic biopsy or surgery prior to anti-PD-1 ICI therapy start; and (vi) available radiological assessment with measurable lesions after the third cycle treatment. Tumor response was assessed using the Response Evaluation Criteria in Solid Tumors version 1.1 (RECIST 1.1). The objective response was determined by complete response (CR) or partial response (PR). Progression-free survival (PFS) and overall survival (OS) were calculated from the first application of anti-PD-1 therapy until disease progression or death.

All patients provided written informed consent for the analysis of FFPE tissue samples. Ethical approval for the study was obtained from the Hospital Ethics Committee of the First Affiliated Hospital of Sun Yat-sen University. This study was conducted in accordance with the guidelines for biomedical research specified in the Declaration of Helsinki.

### Multiplex immunohistochemistry

To assess the expression of prognostic CMGs in GC samples, multiplexed tyramide signal amplification (TSA) immunofluorescence staining was performed using a fluorescence immunohistochemistry kit from TissueGnostics (TG, USA). FFPE sections (3 μm thick) were deparaffinized, rehydrated, and subjected to antigen retrieval using sodium citrate buffer. Endogenous peroxidase was blocked using 3% hydrogen peroxide solution. Primary antibodies against ABCA1 (1:400 dilution, A21976, ABclonal), TSPO (1:200 dilution, A4881, ABclonal), NCEH1 (1:200 dilution, 14021-1-AP, Proteintech), NR1H3 (1:100 dilution, 60134-1-Ig, Proteintech) and HMGCR (1:1000 dilution, 13533-1-AP, Proteintech) were incubated at 4 °C overnight (Additional file 2). The slides were then incubated with HRP Ms & Rb (PR30009, Proteintech) for 20 min at room temperature (RT) before being incubated with TG TSA fluorochromes (TG520N, TG570N, TG620N, TG650N and TG700N) for 10 min at RT. Antigen retrieval with sodium citrate buffer was performed between rounds of tyramide signal amplification to prevent cross-reactivity. Finally, the slides were counterstained with DAPI for 10 min. Image acquisition was performed using TissueFAXS Spectra (TG).

The fluorescence intensity of each marker was quantified using Strataquest software (TG). Positive cells were identified using the 'Cell Masks' or 'Nucleus Masks' algorithm in Strataquest software. The positive cell ratio was calculated with the same fluorescence intensity and area thresholds. Finally, the above correlation coefficients and positive cell ratio were used to calculate the risk score of each sample.

### Statistical analysis

All statistical analyses were performed using R software (version 4.1.1). Student’s t test or the Wilcoxon test was utilized to compare differences in continuous variables between two groups, while the chi-square test was applied to compare categorical variables. To assess the prognostic significance of CMGs and the risk model, we used the Cox proportional hazards model [35], and survival curves were generated using the Kaplan‒Meier method. The R package 'survminer' was used to obtain the optimal cutoff value for survival analysis. Correlation tests were performed using Spearman correlation analysis. To control for false discovery rates, we used the Benjamini‒Hochberg method to adjust the P value. Statistical significance was defined as a P value less than 0.05, and all tests were two-sided.

## Result

### Expression of CMGs in GC

**Fig. 1:**
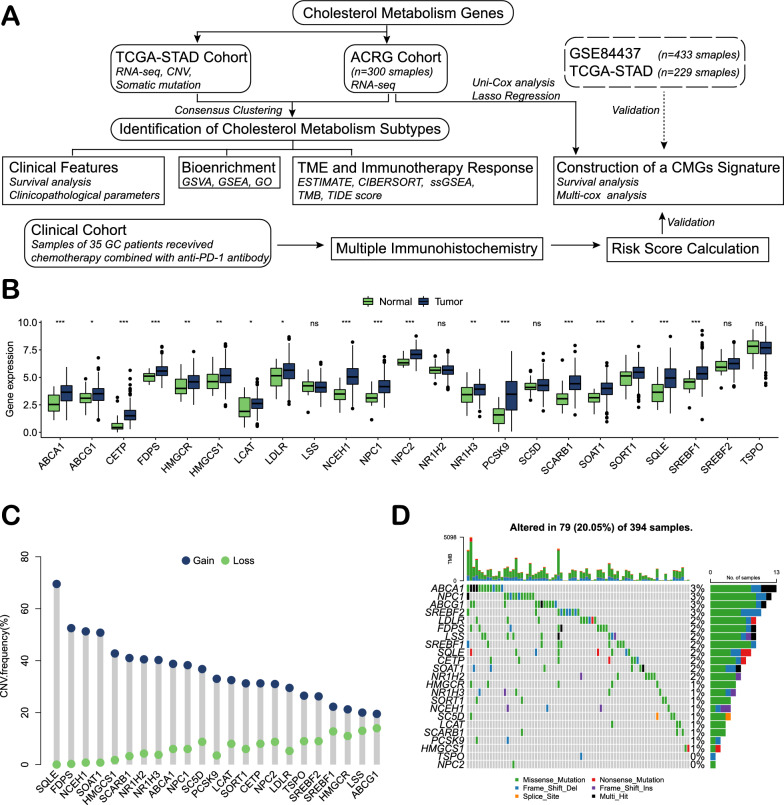
Expression of cholesterol metabolism genes in gastric cancer. **A** The flow chart of this study. **B** RNA levels of the 23 cholesterol metabolism genes in 375 gastric cancer tissues and 32 normal tissues from the TCGA-STAD cohort. **C** The CNV frequency of 23 cholesterol metabolism genes in the TCGA-STAD cohort. **D** The mutation frequency of 23 cholesterol metabolism genes in 394 patients with gastric cancer from the TCGA-STAD cohort

The workflow of this study is shown in Fig. 1A. A total of 23 CMGs (ABCA1, ABCG1, CETP, FDPS, HMGCR, HMGCS1, LCAT, LDLR, LSS, NCEH1, NPC1, NPC2, NR1H2, NR1H3, PCSK9, SC5D, SCARB1, SOAT1, SORT1, SQLE, SREBF1, SREBF2 and TSPO) were retrieved from public databases, and their expression levels were compared between 375 GC tissues and 32 normal gastric tissues (Fig. 1B). CNV frequency analysis revealed a prevalent CNV alteration in 23 CMGs, and all of them were focused on copy number amplification (Fig. 1C). Somatic mutation analysis showed that 79 out of 394 samples (20.05%) contained CMG mutations. Among these, ABCA1 (3%), NPC1 (3%), ABCG1 (3%) and SREBF2 (3%) exhibited the highest gene mutation rates (Fig. 1D).

### Different cholesterol metabolism patterns in GC

**Fig. 2:**
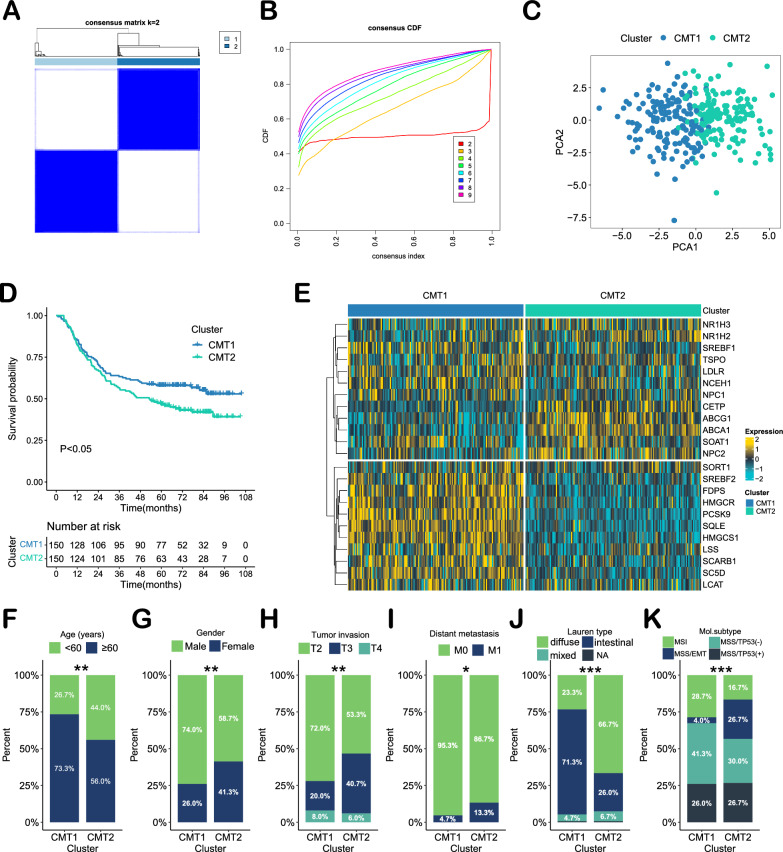
Identify different cholesterol metabolism patterns in 300 patients with gastric cancer from the ACRG cohort. **A** The consensus matrix's heatmap of two clusters (k = 2). **B** The consensus matrix's CDF plot from k = 2–9. **C** Principal component analysis (PCA) of two subtypes. **D** Survival analysis of cholesterol metabolism subtypes based on OS (log-rank test). **E** Heatmap of cholesterol metabolism subtypes defined in the ACGR cohort. **F**‒**K** Comparison of clinicopathological parameters and molecular subtypes in patients with two subtypes of gastric cancer. *P < 0.05, **P < 0.01, ***P < 0.001, ****P < 0.0001

Cluster analysis divided samples from the ACRG dataset into two distinct clusters: cholesterol metabolism type 1 (CMT1) and cholesterol metabolism type 2 (CMT2) (Fig. 2A, B). Principal component analysis (PCA) showed a high degree of differentiation between the two subtypes (Fig. 2C). Survival analysis revealed that CMT2 was associated with a worse prognosis than CMT1 (P < 0.05, Fig. 2D). The heatmap displays the differences in CMG expression between CMT1 and CMT2 (Fig. 2E). CMT1 had high expression of regulatory factors for cholesterol biosynthesis, such as HMGCR, HMGCS1, SREBF2, SC5D, LSS, SQLE and FDPS. In contrast, cholesterol transporters such as ABCA1, ABCG1, LXRs (NR1H3/2) and NPC2 were highly expressed in CMT2. Thus, based on the function of these genes, CMT1 was characterized by active cholesterol synthesis, and CMT2 was characterized by abnormal cholesterol transport. Additionally, compared to CMT1, CMT2 had younger females, deeper T staging, a higher rate of distant metastasis, a higher prevalence of diffuse-Lauren type, and a higher prevalence of MSS/EMT molecular subtypes, suggesting a worse clinical outcome (Fig. 2F‒K).

To assess the reproducibility of the cholesterol metabolism subtypes, we performed cluster analysis for 229 samples from the TCGA-STAD dataset (excluding patients lacking OS data) using the 23 CMGs and identified two distinct subtypes (Additional file 1: Fig. S1A‒C). In this validation set, CMT2 had similar expression patterns for CMGs and exhibited more GS molecular subtypes, fewer MSI molecular subtypes and a trend toward poorer prognosis (Additional file 1: Fig. S1D‒F).

### Differences in biological characteristics between cholesterol metabolism subtypes

**Fig. 3:**
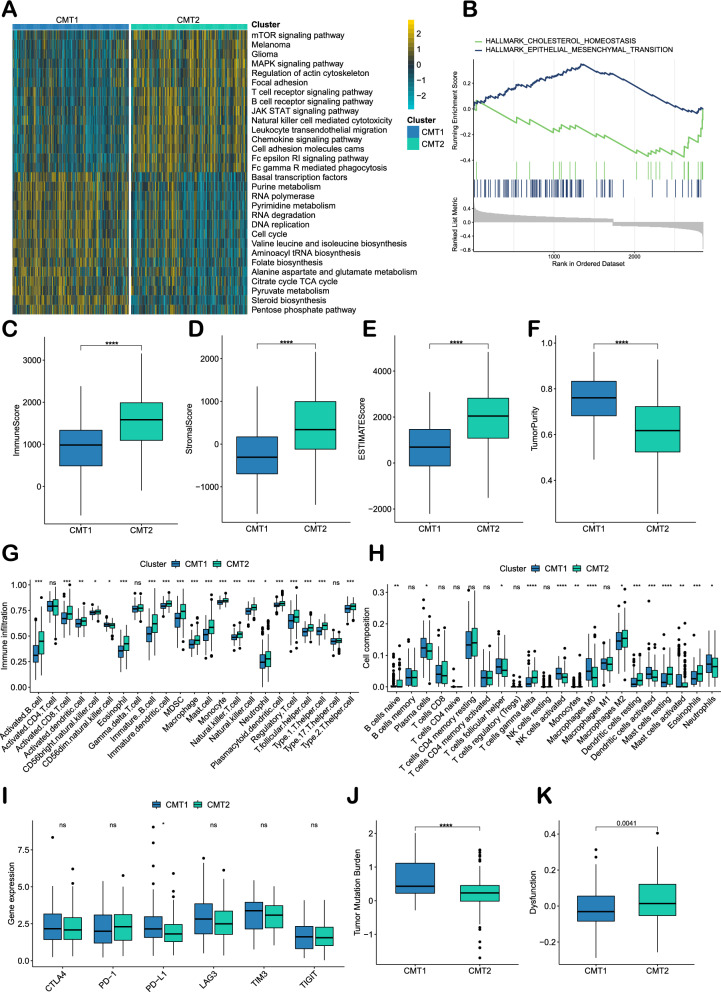
Bioenrichment, immune cell infiltration and immunotherapy efficacy prediction of two cholesterol metabolic subtypes. **A** Gene Set Variation Analysis (GSVA) of two subtype-related pathways in the ACRG cohort. **B** Gene Set Enrichment Analysis (GSEA) of differential genes of two subtypes in the ACRG cohort (CMT2 versus CMT1). **C**‒**F** Comparison of the ESTIMATE score, Immune score, Stromal score and tumor purity of the two subtypes in the ACRG cohort (Wilcoxon test). **G** Comparison of immune cell infiltration between the two subtypes in the ACRG cohort (ssGSEA, Wilcoxon test). **H** The proportions of infiltrating immune cells in the two subtypes were determined using the CIBERSORT tool. **I** Expression levels of immune checkpoint genes in the TCGA-STAD cohort. **J** Tumor mutation burden in the two subtypes in the TCGA-STAD cohort. **K** TIDE scores in the two subtypes in the TCGA-STAD cohort. *P < 0.05, **P < 0.01, ***P < 0.001, ****P < 0.0001

We obtained insights into the biological differences between the two GC subtypes by GSVA. CMT1 was closely related to the cell cycle and transcription and was active in cholesterol biosynthesis. However, CMT2 was enriched in pathways including (1) malignant tumors such as melanoma and glioma; (2) pathways related to tumor progression, such as the mTOR signaling pathway, MAPK signaling pathway and JAK-STAT signaling pathway; and (3) immune cell infiltration and cell adhesion (Fig. 3A). Differential expression analysis of the two GC subtypes identified 2854 DEGs (Additional file 1: Fig. S2A). Additional file 1: Fig. S2B shows the results of the GO analysis. GSEA revealed that CMT2 had upregulation of the epithelial‒mesenchymal transition (EMT) pathway and downregulation of the cholesterol metabolic homeostasis pathway when compared to CMT1 (Fig. 3B).

### Correlation between CMT and immune cell infiltration and immunotherapy response in GC

ESTIMATE analysis demonstrated that the distributions of the ESTIMATE score, Immune score, and Stromal score were higher in CMT2 tumors, whereas the tumor purity was lower (Fig. 3C‒F). Further analysis using ssGSEA revealed significant differences in the degree of immune cell infiltration between the two subtypes and showed more abundant immune-infiltrating cells in CMT2 (Fig. 3G). The results of CIBERSORT showed the proportions of different types of immune cells. Naïve B cells, gamma delta T cells, M2-like macrophages, resting dendritic cells, resting mast cells and eosinophils were more abundant in CMT2 GC than in CMT1 GC. In contrast, CMT2 exhibited lower levels of infiltration of plasma cells, follicular helper T cells, activated NK cells, M0-like macrophages, activated dendritic cells, activated mast cells and neutrophils (Fig. 3H). In summary, although there was a higher degree of immune cell infiltration in CMT2 GC, naïve, inactive and suppressive immune cells occupied the TME. Using data from the TCGA-STAD cohort, we compared the expression profiles of NK cell-related active and inhibitory receptors [36]. The expression of NK cell activated receptor NKp30 was higher in CMT2, while the expression of CD16 was lower (Additional file 1: Fig. S3A). Inhibitory receptors KLRB1 and KLRG1 were highly expressed in CMT2 (Fig. 3B). For the related ligands and factors of NK cells in the immune microenvironment, the expressions of NKG2DL (MICA, MICB, ULBP2, ULBP3, ULBP6), PD-L1, CD155, AMDAM10/17, and IL-10 were significantly lower in CMT2 (Additional file 1: Fig. S3C).

Further, we explored the association between immunotherapy response and CMT. Compared to CMT1, there was no significant difference in expressions of CTLA4, PD-1, LAG3, TIM3 and TIGIT in CMT2; however, CMT2 had a lower expression of PD-L1, a lower TMB and a higher Tide score (Fig. 3I‒K). These results suggested that CMT2 GC was less responsive to anti-PD-1 immunotherapy.

### Identification of prognostic CMGs and construction of risk model

**Fig. 4:**
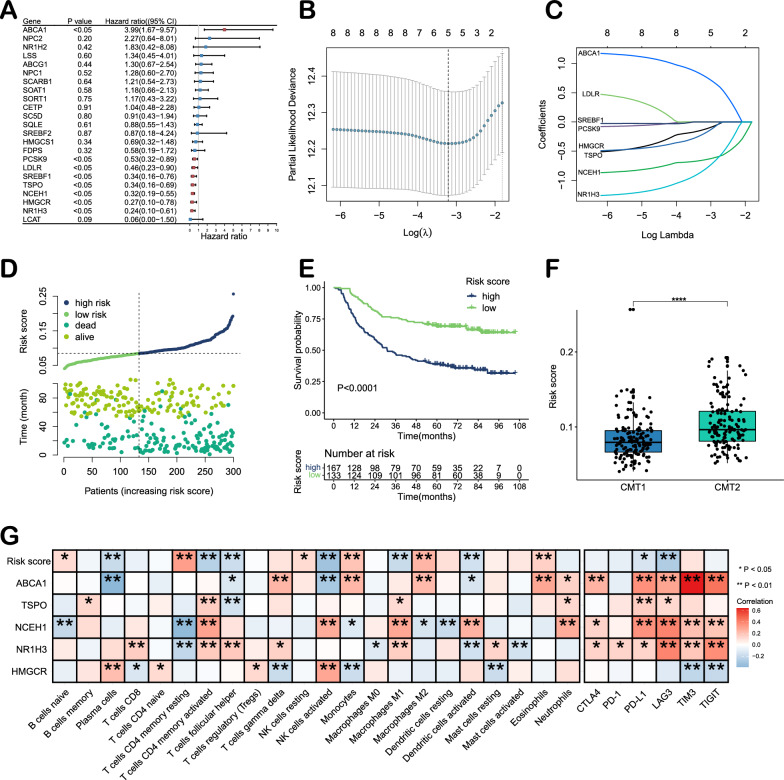
Construction of a gene risk score model in the ACRG cohort. **A** Prognostic analyses for 23 cholesterol metabolism genes using univariate Cox regression model. **B** Screening of prognostic model genes using LASSO regression. **C** Cross-validation of LASSO regression parameter selection. **D** Distribution of the risk score and survival status of 300 patients in the ACRG cohort. **E** Survival analysis of high- and low-risk patients based on OS (log-rank test). **F** Risk scores of CMT1 and CMT2 (Wilcoxon test). **G** Correlation heatmap of the risk score and prognostic model genes with immune cell infiltration and immune checkpoint genes

Univariate Cox regression analysis identified 8 CMGs significantly associated with the prognosis of GC patients (P < 0.05, Fig. 4A). LASSO regression analysis further selected prognostic signature genes, and five CMGs (ABCA1, TSPO, NCEH1, NR1H3 and HMGCR) were used to construct a predictive model based on the risk score (Fig. 4B, C). The risk score for each patient was calculated using the following formula: Risk score = (0.775 × ABCA1) + (− 0.830 × NR1H3) + (− 0.132 × TSPO) + (− 0.653 × NCEH1) + (− 0.205 × HMGCR). Based on the optimal cutoff obtained using the R package 'survminer', patients were divided into high- (n = 167) and low-risk (n = 133) groups (Fig. 4D). The high-risk group exhibited a significantly higher death rate than the low-risk group (P < 0.001, Fig. 4E). Multivariate analysis showed that the risk score was an independent risk factor (P < 0.001, Additional file 1: Table S2). For TCGA-STAD and GSE84437, we calculated the risk score of patients using the above formula. The results indicated that the prognosis of the high-risk group was worse than that of the low-risk group in those validation sets (Additional file 1: Fig. S4).

The risk score was significantly higher in CMT2 (Fig. 4F). The correlation heatmap revealed the relationship between the CMG risk score and the immune microenvironment. There were negative correlations between the risk score and plasma cells, activated CD4 memory T cells, follicular helper T cells, activated NK cells, M1-like macrophages, activated dendritic cells, and immune checkpoints (PD-L1 and LAG3). In contrast, the risk score was positively correlated with naïve B cells, resting CD4 memory T cells, resting NK cells, monocytes, M2-like macrophages, resting dendritic cells, resting activated mast cells and eosinophils (Fig. 4G).

### CMG risk score was correlated with response to anti-PD-1 therapy

**Fig. 5:**
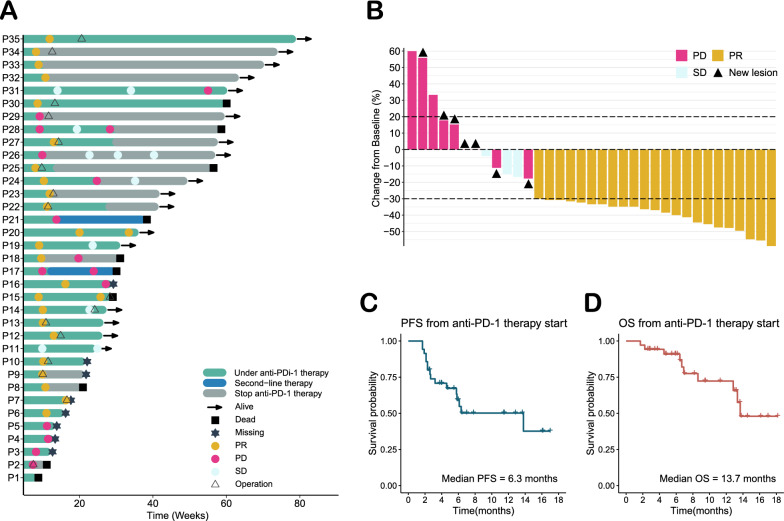
Information on anti-PD-1 treatment in the clinical cohort. **A** Swimmer plot of 35 gastric cancer patients. **B** Radiological evaluation of 35 gastric cancer patients receiving three cycles of chemotherapy plus anti-PD-1 therapy. **C** PFS from anti-PD-1 therapy start in months. **D** OS from anti-PD-1 therapy starting in months

To further verify the role of our five-CMG signature (ABCA1, TSPO, NCEH1, NR1H3 and HMGCR) in predicting the response to immunotherapy, we retrospectively enrolled 35 GC patients treated with a PD-1 inhibitor. In this cohort, the average age of the 35 patients (9 women, 25.7%; 26 men, 74.3%) was 58 (range 24‒78) years. The cohort included 4 stage 2 patients, 9 stage 3 patients, and 22 stage 4 patients, most of whom were poorly differentiated (30/35, 85.7%). The median number of anti-PD-1 applications was 3 (2‒11). Thirty-three patients (94.3%) received basal chemotherapy with SOX (S-1 plus oxaliplatin), 1 patient received CapeOX (capecitabine plus S-1), and 1 patient with EGJ received paclitaxel plus carboplatin. The median follow-up of the cohort was 6.9 months (1.7‒18.2 months) (Table 1). The swimmer plot summarized the treatment of the 35 GC patients (Fig. 5A). After 3 cycles of anti-PD-1 therapy, 23 out of 35 (65.7%) patients achieved a PR, 3 out of 35 (8.7%) patients achieved SD, and 9 out of 35 (25.6%) patients achieved PD (Fig. 5B). The median PFS and OS from the start of anti-PD-1 therapy were 6.3 (1.7‒17.0) and 13.7 (1.7‒18.2) months, respectively (Fig. 5C, D).

**Table 1:** Clinical Information of the validation cohort

Characteristic	*N* = *35*
Age [*M* (range), year]	58 (24–78)
Gender [n (%)]	
Male	26 (74.29)
Female	9 (25.71)
Location [n (%)]	
Upper 1/3	12 (34.29)
Middle 1/3	10 (28.57)
Lower 1/3	10 (28.57)
Whole	1 (2.86)
Remnant	2 (5.71)
Tumor invasion [n (%)]	
T2	4 (11.43)
T3	4 (11.43)
T4a	19 (54.28)
T4b	8 (22.86)
Lymph node [n (%)]	
N0	1 (2.86)
N1–3	34 (97.14)
Distant metastasis [n (%)]	
M0	17 (48.57)
M1	18 (51.43)
cTNM stage [n (%)]	
2A	3 (8.57)
2B	1 (2.86)
3	9 (25.71)
4A	4 (11.43)
4B	18 (51.43)
Differentiation [n (%)]	
Moderate	5 (14.29)
Poor	30 (85.71)
Anti-PD-1 agent [n (%)]	
Camrelizumab	11 (31.43)
Tislelizumab	1 (2.86)
Pembrolizumab	3 (8.57)
Nivolumab	8 (22.85)
Sintilimab	12 (34.29)
Received cycles of anti-PD-1 therapy [*M* (range)]	3 (2–11)
Chemotherapy [n (%)]	
SOX	33 (94.28)
CapeOx	1 (2.86)
Paclitaxel plus carboplatin	1 (2.86)
Follow-up time [*M* (range), months]	6.9 (1.7–18.2)
PFS [*M* (range), months]	6.3 (1.7–17.0)
OS [*M* (range), months]	13.7 (1.7–18.2)

*M* median, *SOX* S-1 plus Oxaliplatin, *CapeOx* capecitabine plus Oxaliplatin, *PFS* progression free survival, *OS* overall survival

**Fig. 6:**
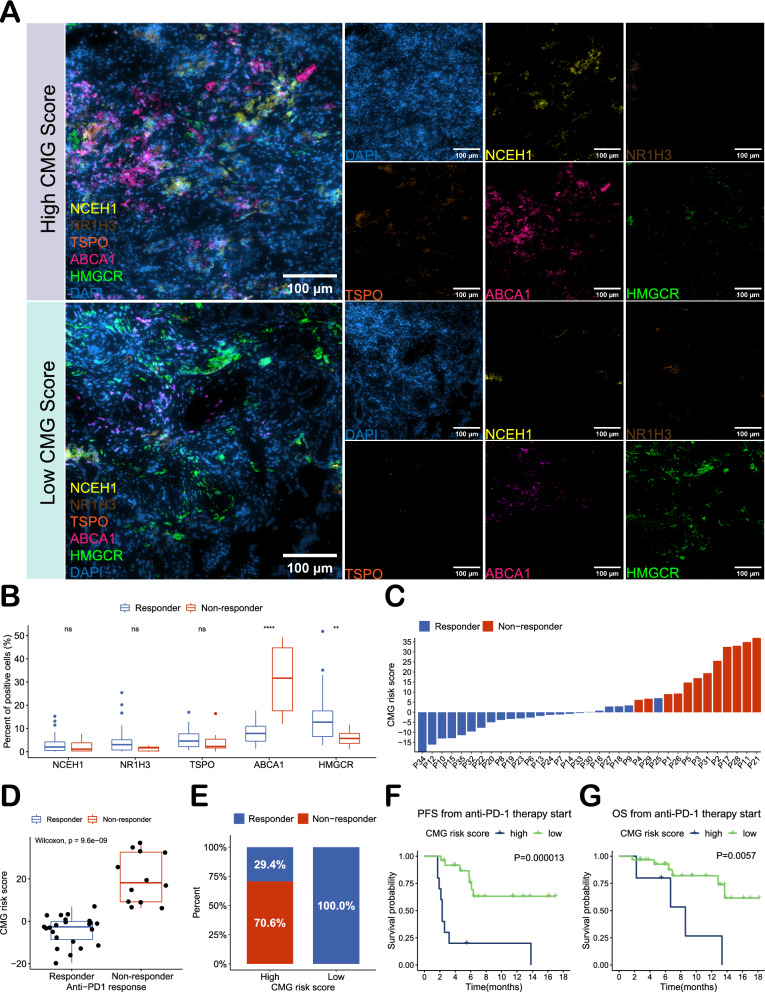
Validation in 35 clinical samples using multiplex immunohistochemistry. **A** Representative stained images of high- and low-risk groups. **B** Expression of NCEH1, NR1H3, TSPO, ABCA1 and HMGCR in responders and non-responders. **C**, **D** Risk scores in responders and non-responders. **E** Proportion of responders/non-responders in the high- and low-risk groups (using the median number for the cutoff value). **F** Comparison of PFS and OS (**G**) between high- and low-risk patients (using optimum cutoff value). *P < 0.05, **P < 0.01, ***P < 0.001, ****P < 0.0001

A panel of multiplex immunohistochemical staining (NCEH1, NR1H3, TSPO, ABCA1 and HMGCR) was performed and used to calculate the CMG risk score for each patient. Figure 6A shows representative staining images. The expression levels of NCEH1, NR1H3 and TSPO were not significantly different between the two groups (Wilcoxon test, P > 0.05). However, the non-responders had higher ABCA1 expression and lower HMGCR expression (Wilcoxon test, P < 0.0001 and P < 0.01, respectively; Fig. 6B) and therefore a higher CMG risk score (Wilcoxon test, P < 0.0001; Fig. 6C, D). The 35 patients were divided into high- and low-risk groups by median CMG risk score, and patients in the high-risk group had a lower response rate (33.3% vs 100.0%; Fig. 6E). Based on the optimal cutoff value, the high CMG risk group had shorter PFS and OS (log-rank test, P < 0.0001 and P = 0.0057, respectively; Fig. 6F, G).

## Discussion

Cholesterol metabolism has garnered increasing attention in cancer research, attributed to its pivotal role in cancer prevention and treatment [37]. Dysregulation of cholesterol metabolism has been linked to cancer development, influencing crucial aspects such as proliferation, invasion, immune cell function, and chemotherapy sensitivity [38]. The growing body of research underscores the importance of cholesterol metabolism in tumor immunity [39]. Therefore, amid numerous pathways, we chose to investigate the cholesterol metabolism pathway. Rigorous gene selection and iterative data analysis calculations contributed to the results we present in our study. In terms of the mechanism of cholesterol reprogramming, abnormalities in cholesterol synthesis or cholesterol transport have been proven in numerous studies [40]. However, previous studies have often focused on the roles of specific genes in cholesterol synthesis or cholesterol transport. To the best of our knowledge, no study has focused on both cholesterol synthesis and cholesterol transport at the same time. It is still unknown whether cholesterol synthesis and cholesterol transport are different in influencing the development of human cancer. In this study, we explored the variation in cholesterol metabolism patterns in GC. Based on representative cholesterol metabolism genes and their distinct expression patterns, we classified GC into two cholesterol metabolic subtypes, CMT1 and CMT2. Among the cholesterol metabolism genes we selected, HMGCR, HMGCS1, LSS, SQLE and FDPS encode enzymes in the cholesterol biosynthesis pathway, and SREBF2 can activate the transcription of these genes [12, 41]. Due to the high expression of the above genes, CMT1 is defined as GC with active cholesterol synthesis. Similarly, ABCA1 and ABCG1 remove cholesterol from cells under the regulation of LXRs, and we observed that these genes are highly expressed in CMT2 GC, which is defined as GC with abnormal cholesterol transport.

We performed a multi-platform analysis of the two identified cholesterol metabolic subtypes, suggesting that abnormal cholesterol transport has a negative effect on GC. Stroma activation and tumor immunosuppression are widely studied features of malignant tumors [42, 43], which also characterize CMT2 GC, as evidenced by the enrichment of the EMT pathway [44]. The increased stromal score and decreased tumor purity in CMT2, as indicated by the ESTIMATE algorithm, further support the activation of stromal components. Moreover, while CMT2 showed higher immune cell infiltration, the presence of M2-like macrophages may contribute to the immunosuppressive tumor microenvironment [45, 46]. Cholesterol has been implicated in the activation of Toll-like receptors (TLRs) on macrophages, leading to chronic inflammation within the tumor microenvironment, thereby promoting tumor progression [47]. Therefore, the polarization of macrophages toward the M2 type may be a potential mechanism underlying the negative effects of abnormal cholesterol transport, which warrants further investigation.

To simplify our classification model and facilitate clinical application, we identified a 5-gene prognostic signature, ABCA1, NR1H3, TSPO, NCEH1, and HMGCR, and validated it using clinical specimens. The roles of these genes in GC remain unclear. For instance, ABCA1 is typically studied as a cancer suppressor due to its role in reducing intracellular cholesterol levels [48, 49]. However, in recent years, many studies have found that its expression is positively correlated with poor tumor prognosis and chemotherapy resistance [50–52]. In our study, it was associated with a poor prognosis in GC. Considering its central role in cholesterol transport, ABCA1 could represent dysregulation of cholesterol transport activity in GC. Notably, its role in macrophage polarization was demonstrated [53], which is consistent with the up-regulation of M2 macrophage infiltration in CMT2 GC. In line with our results, NR1H3 (LXRα) has also been classified as a cancer suppressor in numerous studies [54]. TSPO is a receptor involved in the regulation of cellular proliferation, apoptosis, and mitochondrial functions [55, 56]. Few studies have been conducted on TSPO in GC, and its mRNA level has been found to have limited prognostic value [57]. NCEH1 plays an initial role in converting cholesterol esters into free cholesterol, and its studies in cancer are also limited. Overexpression of NCEH1 has been associated with breast cancer, ovarian cancer, and other cancer types, but its relationship with GC has not been established [58–60].

Despite the unclear roles of these genes in GC, our results demonstrate that they collectively represent the extent of abnormal cholesterol transport in GC. The risk model based on these genes aligned well with CMT2 GC and correlated with immunotherapy response in our clinical cohort. These results suggest that our 5-CMG signature is an effective biomarker for immunotherapy response and prognosis in GC. To the best of our knowledge, our study is the first to establish a link between cholesterol metabolism and immunotherapy in GC.

While our study has provided valuable insights, several limitations should be acknowledged. First, the validation sample size was relatively small, the follow-up time was short, and the results should be confirmed in larger cohorts. Second, the retrospective nature of the study design may have introduced selection bias and limited the scope of detailed clinical and pathological analysis. Finally, the underlying mechanisms driving these associations were not elucidated. Further research is warranted to explore the precise mechanisms through which cholesterol metabolism genes influence GC.

## Conclusion

Our study reveals two distinct cholesterol metabolic subtypes of GC: CMT1 characterized by active cholesterol biosynthesis and CMT2 characterized by abnormal cholesterol transport. CMT2 is associated with worse prognosis and reduced response to immunotherapy. Genetic signature based on patterns of cholesterol metabolism serve as an effective biomarker for prognosis and response to anti-PD-1 therapy in GC. These findings shed light on the role of cholesterol metabolism in GC and its potential implications for personalized treatment strategies.

### Supplementary Information

**Additional file 1:** **Figure S1**. Validation the two cholesterol metabolism patterns in 229 patients with gastric cancer from TCGA-STAD cohort. **Figure S2**. Differential expression gene (DEG) screening of two subtypes and GO enrichment analysis in ACRG cohort. **Figure S3**. The expression profiles of NK cells receptors and related ligands of two cholesterol metabolic subtypes. **Figure S4**. Validation of risk score model in TCGA-STAD cohort and GSE84437 cohort. **Table S1**. List of Cholesterol Metabolism Genes.

**Additional file 2.** Information of primary antibodies in multiple immunohistochemistry.

## Data Availability

The data that support the findings of our study are available from the corresponding author upon reasonable request. The public data used in our study can be obtained from The Cancer Genome Atlas (TCGA) database (https://cancergenome.nih.gov/) and NCBI Gene Expression Omnibus (GEO) database (https://www.ncbi.nlm.nih.gov/geo/).
