# Supplementary material for: Multiplex immunohistochemistry defines two cholesterol metabolism patterns predicting immunotherapeutic outcomes in gastric cancer
Source: J Transl Med. 2023 Dec 7;21:887. doi: 10.1186/s12967-023-04758-4 (PMC10702056; doi:10.1186/s12967-023-04758-4)
Supplement: Supplementary file 1 — Additional file 1: Figure S1. Validation the two cholesterol metabolism patterns in 229 patients with gastric cancer from TCGA-STAD cohort. Figure S2. Differential expression gene (DEG) screening of two subtypes and GO enrichment analysis in ACRG cohort. Figure S3. The expression profiles of NK cells receptors and related ligands of two cholesterol metabolic subtypes. Figure S4. Validation of risk score model in TCGA-STAD cohort and GSE84437 cohort. Table S1. List of Cholesterol Metabolism Genes. [file 12967_2023_4758_MOESM1_ESM.pdf]

Additional file 1

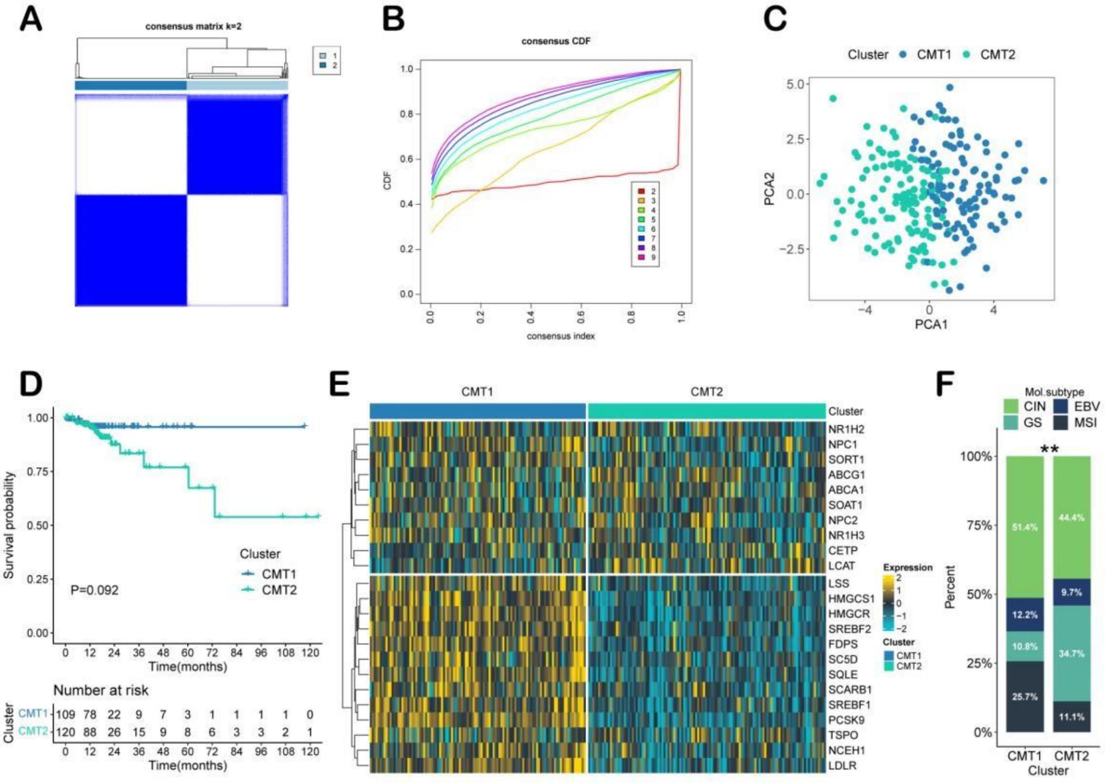

Figure S1: Validation the two cholesterol metabolism patterns in 229 patients with gastric cancer from TCGA-STAD cohort. (A) The consensus matrix's heatmap of two clusters (k=2). (B) The consensus matrix's CDF plot from k=2 to 9. (C) Principal component analysis (PCA) of two subtypes. (D) Survival analysis of cholesterol metabolism subtypes based on Overall survival (log-rank test). (E) Heatmap of cholesterol metabolism subtypes defined in TCGA-STAD cohort. (f) Comparison of the TCGA molecular subtypes in patients with two subtypes of gastric cancer. *P < 0.05, **P<0.01, ***P < 0.001.

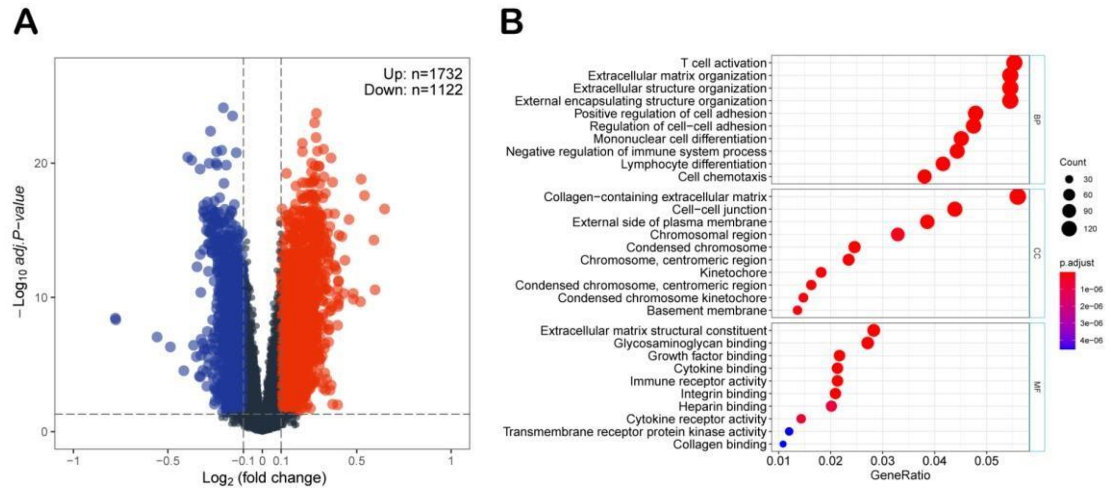

Figure S2: Differential expression gene (DEG) screening of two subtypes and GO enrichment analysis in ACRG cohort. (A)

Volcano plot of differential expression analysis using limma package (CMT2 versus CMT1). (B) GO enrichment analysis

(Biological process, Cellular component, and Molecular function) of DEGs.

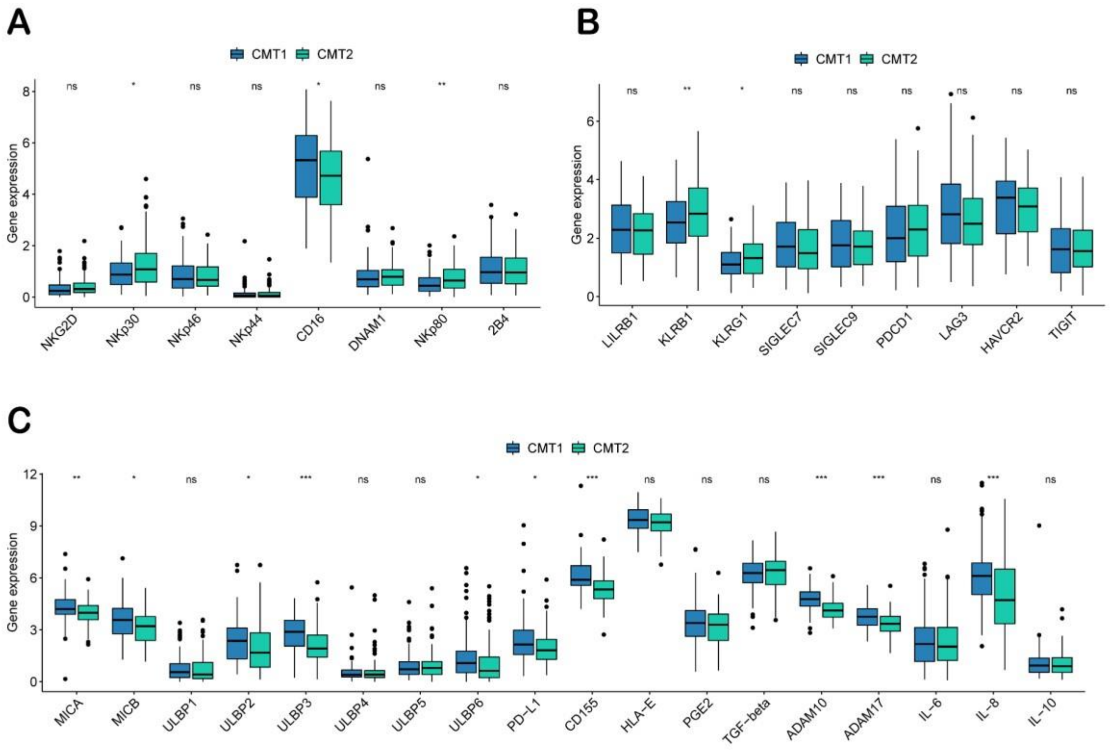

Figure S3: The expression profiles of NK cells receptors and related ligands of two cholesterol metabolic subtypes. (A) Comparison of NK cell activating receptors between CMT1 and CMT2. (B) Comparison of NK cell inhibitory receptors between CMT1 and CMT2. (C) Comparison of NK cell related ligands and molecules in the TME between CMT1 and CMT2. *P < 0.05, **P<0.01, ***P < 0.001.

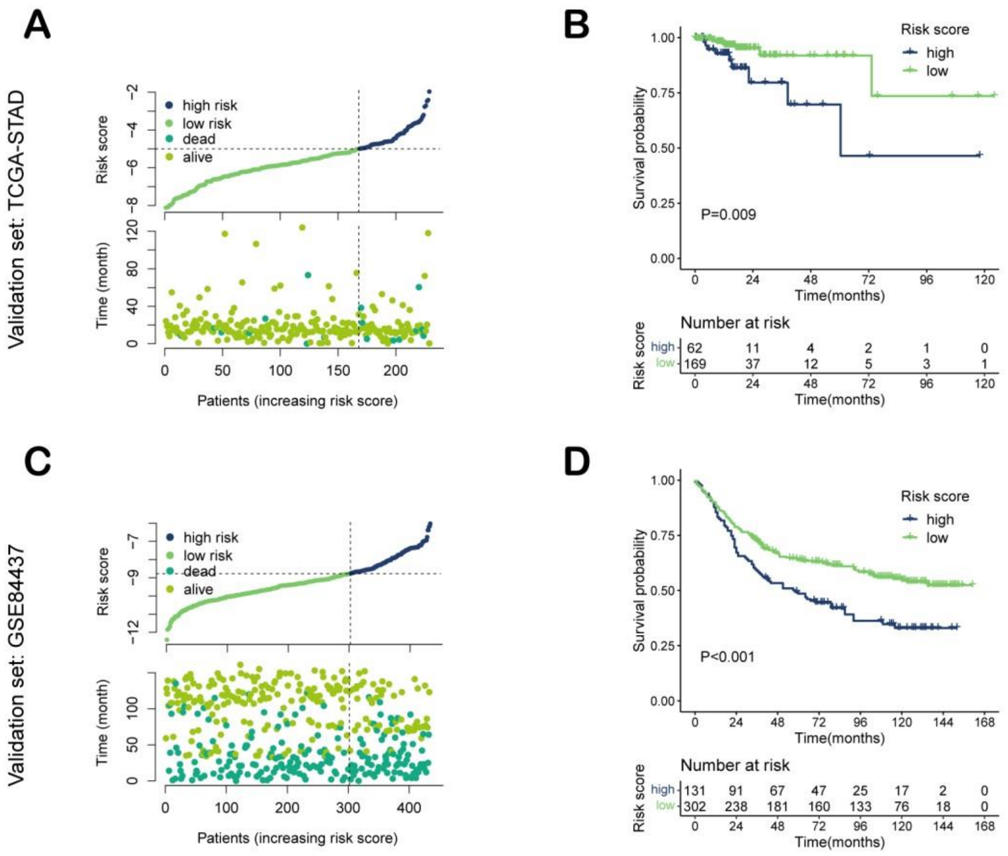

Figure S4: Validation of risk score model in TCGA-STAD cohort and GSE84437 cohort. (A) Distribution of risk score and survival status of 229 patients in TCGA cohort. (B) Survival analysis of high- and low risk patients in TCGA cohort (log-rank test). (C) Distribution of risk score and survival status of 433 patients in GSE84437 cohort. (D) Survival analysis of high- and low risk patients in GSE84437 cohort (log-rank test).

Table S1 List of Cholesterol Metabolism Genes

Gene Name	Full Name	Category	NCBI Entrez ID
ABCA1	ATP Binding Cassette Subfamily A Member 1	Protein Coding	19
ABCG1	ATP Binding Cassette Subfamily G Member 1	Protein Coding	9619
CETP	Cholesteryl Ester Transfer Protein	Protein Coding	1071
FDPS	Farnesyl Diphosphate Synthase	Protein Coding	2224
HMGCR	3-Hydroxy-3-Methylglutaryl-CoA Reductase	Protein Coding	3156
HMGCS1	3-Hydroxy-3-Methylglutaryl-CoA Synthase 1	Protein Coding	3157
LCAT	Lecithin-Cholesterol Acyltransferase	Protein Coding	3931
LDLR	Low Density Lipoprotein Receptor	Protein Coding	3949
LSS			
NCEH1	Neutral Cholesterol Ester Hydrolase 1	Protein Coding	57552
NPC1	Niemann-Pick C1 protein	Protein Coding	4864
NPC2	NPC Intracellular Cholesterol Transporter 2	Protein Coding	10557
NR1H2	Nuclear Receptor Subfamily 1 Group H Member 2	Protein Coding	7376
NR1H3	Nuclear Receptor Subfamily 1 Group H Member 3	Protein Coding	10062
PCSK9	Proprotein Convertase Subtilisin/Kexin Type 9	Protein Coding	255738
SC5D	Sterol-C5-Desaturase	Protein Coding	6309
SCARB1	Scavenger Receptor Class B Member 1	Protein Coding	949
SOAT1	Sterol O-Acyltransferase 1	Protein Coding	6646
SORT1	Sortilin 1	Protein Coding	6272
SQLE	Squalene Epoxidase	Protein Coding	6713
SREBF1	Sterol Regulatory Element Binding Transcription Factor 1	Protein Coding	6720

SREBF2	Sterol Regulatory Element Binding Transcription Factor 2	Protein Coding	6721
TSPO	Translocator Protein	Protein Coding	706

Data source: GeneCards: The Human Gene Database (https://www.genecards.org/)

Table S2 Univariate and Multivariate Cox Regression Analysis for Patients from the ACRG Cohort

Factors	Univariate		Multivariate	
	Hazard ratio (95% CI)	P value	Hazard ratio (95% CI)	P value
Age	1.01(1.00-1.03)	0.181		
Gender<br>Male vs Female	0.90(0.65-1.27)	0.559		
T stage<br>T3-T4 vs T2	2.40(1.74-3.30)	<0.001	1.88(1.36-2.59)	<0.001
N stage<br>N1-N3 vs N0	2.81(1.43-5.53)	0.003	2.64(1.34-5.19)	0.005

M stage<br>M1 vs M0	3.84(2.48-5.94)	<0.001	3.48(2.24-5.42)	<0.001
CMG risk score	1.08(1.05-1.11)	<0.001	1.06(1.03-1.09)	<0.001
