# Supplementary material for: Multiplex immunohistochemistry defines two cholesterol metabolism patterns predicting immunotherapeutic outcomes in gastric cancer
Source: J Transl Med. 2023 Dec 7;21:887. doi: 10.1186/s12967-023-04758-4 (PMC10702056; doi:10.1186/s12967-023-04758-4)
Supplement: Supplementary file 2 — Additional file 2. Information of primary antibodies in multiple immunohistochemistry. [file 12967_2023_4758_MOESM2_ESM.pdf]

ABCA1 Rabbit mAb

Catalog No.: A21976 Recombinant

Basic Information

Observed MW
Refer to figures

Calculated MW
254kDa

Category
Primary antibody

Applications
IF/ICC

Cross-Reactivity
Human, Mouse, Rat

CloneNo number
ARC54239

Background

The membrane-associated protein encoded by this gene is a member of the superfamily of ATP-binding cassette (ABC) transporters. ABC proteins transport various molecules across extra- and intracellular membranes. ABC genes are divided into seven distinct subfamilies (ABC1, MDR/TAP, MRP, ALD, OABP, GCN20, White). This protein is a member of the ABC1 subfamily. Members of the ABC1 subfamily comprise the only major ABC subfamily found exclusively in multicellular eukaryotes. With cholesterol as its substrate, this protein functions as a cholesteral efflux pump in the cellular lipid removal pathway. Mutations in both alleles of this gene cause Tangier disease and familial high-density lipoprotein (HDL) deficiency.

Recommended Dilutions

**IF/ICC**	1:50 - 1:200

Immunogen Information

Gene ID
19

Swiss Prot
O95477

Immunogen
Recombinant fusion protein containing a sequence corresponding to amino acids 1170-1350 of human ABCA1 (NP_005493.2).

Synonyms
TGD; ABC1; CERP; ABC-1; HDLDT1; HPALP1; HDLCQTL13

Contact

www.abclonal.com

Product Information

Source
Rabbit

Isotype
IgG

Purification
Affinity purification

Storage
Store at -20℃. Avoid freeze / thaw cycles.
Buffer: PBS with 0.05% proclin300,0.05% BSA,50% glycerol,pH7.3.

Validation Data

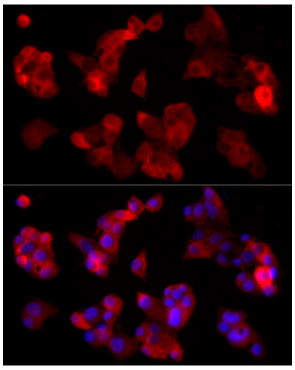

Immunofluorescence analysis of HepG2 using ABCA1 Rabbit mAb (A21976) at dilution of 1:200、1： 1000 (40x lens). Blue: DAPI for nuclear staining.

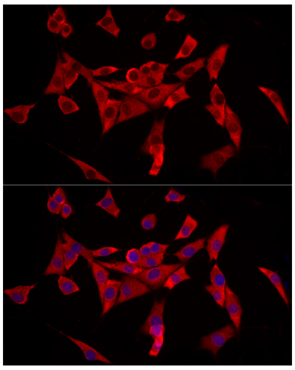

Immunofluorescence analysis of NIH/3T3 using ABCA1 Rabbit mAb (A21976) at dilution of 1:200、1： 1000 (40x lens). Blue: DAPI for nuclear staining.

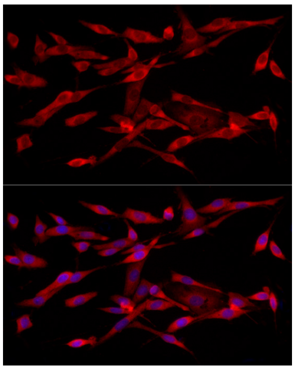

Immunofluorescence analysis of PC-12 using ABCA1 Rabbit mAb (A21976) at dilution of 1:200、1： 1000 (40x lens). Blue: DAPI for nuclear staining.

For research use only. Not for therapeutic or diagnostic purposes.
Please visit http://abclonal.com for a complete listing of recommended products.

For Research Use Only

HMGCR Polyclonal antibody

Catalog Number:13533-1-AP Featured Product 40 Publications

proteintech® Antibodies | ELISA kits | Proteins
www.ptglab.com

Basic Information

Catalog Number:
13533-1-AP

Size:
300 μg/ml

Source:
Rabbit

Isotype:
IgG

Immunogen Catalog Number:
AG4444

GenBank Accession Number:
BC033692

GeneID (NCBI):
3156

Full Name:
3-hydroxy-3-methylglutaryl-Coenzyme A reductase

Calculated MW:
888 aa, 95 kDa

Purification Method:
Antigen affinity purification

Recommended Dilutions:
IHC 1:500-1:2000

Applications

Tested Applications:
IHC, ELISA

Cited Applications:
IF, IHC

Species Specificity:
human, mouse, rat

Cited Species:
human, rat, mouse

Note-IHC: suggested antigen retrieval with TE buffer pH 9.0; (*) Alternatively, antigen retrieval may be performed with citrate buffer pH 6.0

Positive Controls:

IHC : human liver cancer tissue, human small intestine tissue, human heart tissue

Background Information

HMGCR(3-hydroxy-3-methylglutaryl-coenzyme A reductase) is also named as HMG-CoA reductase and belongs to the HMG-CoA reductase family. This protein exists as a 97-kDa glycoprotein in the endoplasmic reticulum (ER) and is the rate-determining enzyme of the biosynthesis pathway(PMID:15830340).The ectopic expression of either full-length HMGCR or its novel splice variant promotes dysregulation of the MVA pathway. And the ectopic expression of cHMGCR-FL(60 kDa) and cHMGCR-D13(55 kDa) can be detected in HepG2 cells(PMID:20696928).This protein is also can exsit as a oligomer(PMID:20696928).

Notable Publications

Author	Pubmed ID	Journal	Application
Hai-Yan Wang	34588618	Acta Pharmacol Sin	WB,IHC
Wei Xu	32935679	Food Funct	WB
Liang Wei Wang	31518366	PLoS Pathog	WB

Storage

Storage:
Store at -20°C. Stable for one year after shipment.
Storage Buffer:
PBS with 0.02% sodium azide and 50% glycerol pH 7.3.
Aliquoting is unnecessary for -20°C storage

For technical support and original validation data for this product please contact:
T: 4006900926 E: Proteintech-CN@ptglab.com W: ptgcn.com

This product is exclusively available under Proteintech Group brand and is not available to purchase from any other manufacturer.

Selected Validation Data

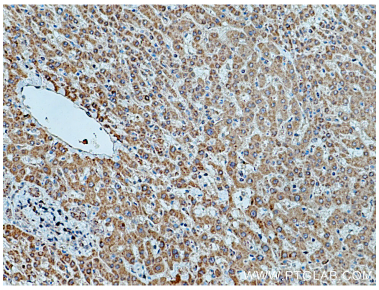

Immunohistochemical analysis of paraffin-embedded human liver cancer tissue slide using 13533-1-AP (HMGCR antibody) at dilution of 1:1000 (under 10x lens). Heat mediated antigen retrieval with Tris-EDTA buffer (pH 9.0).

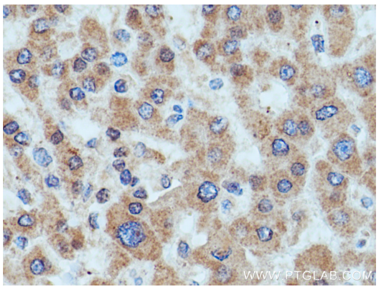

Immunohistochemical analysis of paraffin-embedded human liver cancer tissue slide using 13533-1-AP (HMGCR antibody) at dilution of 1:1000 (under 40x lens). Heat mediated antigen retrieval with Tris-EDTA buffer (pH 9.0).

For Research Use Only

AADACL1 Polyclonal antibody

Catalog Number: 14021-1-AP   1 Publications

proteintech®
Antibodies | ELISA kits | Proteins
www.ptglab.com

Basic Information

Catalog Number:
14021-1-AP

Size:
240 μg/ml

Source:
Rabbit

Isotype:
IgG

Immunogen Catalog Number:
AG5136

GenBank Accession Number:
BC047588

GeneID (NCBI):
57552

Full Name:
arylacetamide deacetylase-like 1

Calculated MW:
46 kDa

Observed MW:
40-50 kDa

Purification Method:
Antigen affinity purification

Recommended Dilutions:
WB 1:1000-1:4000
IP 0.5-4.0 ug for IP and 1:500-1:1000 for WB
IHC 1:20-1:200
IF 1:10-1:100

Applications

Tested Applications:
IF, IHC, IP, WB, ELISA

Cited Applications:
WB

Species Specificity:
human, mouse, rat

Cited Species:
rat

Note-IHC: suggested antigen retrieval with TE buffer pH 9.0; (*) Alternatively, antigen retrieval may be performed with citrate buffer pH 6.0

Positive Controls:

WB : PC-3 cells,

IP : COLO 320 cells,

IHC : human brain tissue, human kidney tissue, human lung tissue, human ovary tissue, human skin tissue, human spleen tissue

IF : COLO 320 cells,

Background Information

AADACL1(Arylacetamide deacetylase-like 1) is also named as NCEH1, KIAA1363 and belongs to the 'GDXG' lipolytic enzyme family. The transmembrane enzyme, AADACL1, controls the production of the monoalkylglycerol ether (MAGE) class of NELs in cancer cells and acts as a 2-acetyl MAGE hydrolase and is likely the principal source for this activity in tumor cells(PMID:21513884). The full length protein has three glycosylation sites and can be N-glycosylated(PMID:19159218). It has 3 isoforms produced by alternative splicing.

Notable Publications

Author	Pubmed ID	Journal	Application
Jingyi Duan	32240813	J Proteomics	WB

Storage

Storage:
Store at -20°C. Stable for one year after shipment.
Storage Buffer:
PBS with 0.02% sodium azide and 50% glycerol pH 7.3.
Aliquoting is unnecessary for -20°C storage

For technical support and original validation data for this product please contact:
T: 4006900926   E: Proteintech-CN@ptglab.com   W: ptgcn.com

This product is exclusively available under Proteintech Group brand and is not available to purchase from any other manufacturer.

Selected Validation Data

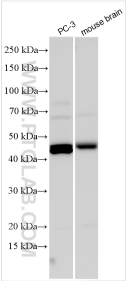

Various lysates were subjected to SDS PAGE followed by western blot with 14021-1-AP (AADACL1 antibody) at dilution of 1:2000 incubated at room temperature for 1.5 hours.

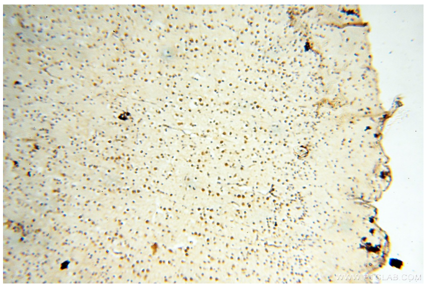

Immunohistochemical analysis of paraffin-embedded human brain using 14021-1-AP (AADACL1 antibody) at dilution of 1:50 (under 10x lens).

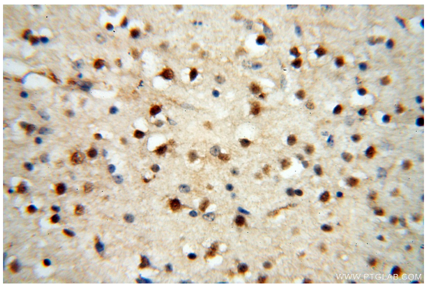

Immunohistochemical analysis of paraffin-embedded human brain using 14021-1-AP (AADACL1 antibody) at dilution of 1:50 (under 40x lens).

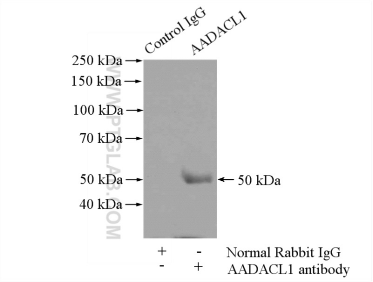

IP Result of anti-AADACL1 (IP:14021-1-AP, 4ug; Detection:14021-1-AP 1:500) with COLO 320 cells lysate 400ug.

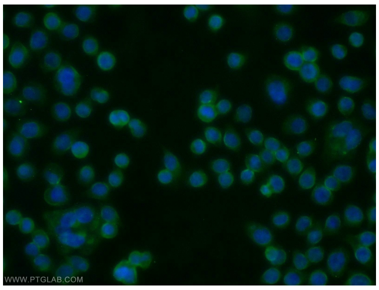

Immunofluorescent analysis of COLO 320 cells using 14021-1-AP (AADACL1 antibody) at dilution of 1:25 and Alexa Fluor 488-conjugated AffiniPure Goat Anti-Rabbit IgG(H+L).

For Research Use Only

NR1H3 Monoclonal antibody

Catalog Number:60134-1-Ig 1 Publications

proteintech®
Antibodies | ELISA kits | Proteins
www.ptglab.com

Basic Information

Catalog Number:
60134-1-Ig

Size:
500 μg/ml

Source:
Mouse

Isotype:
IgG1

Immunogen Catalog Number:
AG6184

GenBank Accession Number:
BC041172

GeneID (NCBI):
10062

Full Name:
nuclear receptor subfamily 1, group H, member 3

Calculated MW:
50 kDa

Observed MW:
50 kDa 44 kDa

Purification Method:
Protein G purification

CloneNo.:
4A4F7

Recommended Dilutions:
WB 1:2000-1:10000
IF 1:10-1:100

Applications

Tested Applications:
IF, WB, ELISA

Cited Applications:
WB

Species Specificity:
human, rat, pig

Cited Species:
mouse

Positive Controls:

WB : T-47D cells, HepG2 cells, MCF-7 cells, COLO 320 cells

IF : HepG2 cells,

Background Information

NR1H3, also known as LXRA, is a liver X receptors, which form a subfamily of the nuclear receptor superfamily and are key regulators of macrophage function, controlling transcriptional programs involved in lipid homeostasis and inflammation. It's also a ligand-activated LXRs and forms heterodimers with retinoid X receptor, then participates in regulating expression of target genes containing LXR response elements.

Notable Publications

Author	Pubmed ID	Journal	Application
Xin Shen	34351342	Food Funct	WB

Storage

Storage:
Store at -20°C. Stable for one year after shipment.
Storage Buffer:
PBS with 0.02% sodium azide and 50% glycerol pH 7.3.
Aliquoting is unnecessary for -20°C storage

For technical support and original validation data for this product please contact:
T: 4006900926 E: Proteintech-CN@ptglab.com W: ptgcn.com

This product is exclusively available under Proteintech Group brand and is not available to purchase from any other manufacturer.

Selected Validation Data

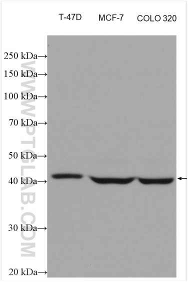

Various lysates were subjected to SDS PAGE followed by western blot with 60134-1-Ig (NR1H3 antibody) at dilution of 1:5000 incubated at room temperature for 1.5 hours.

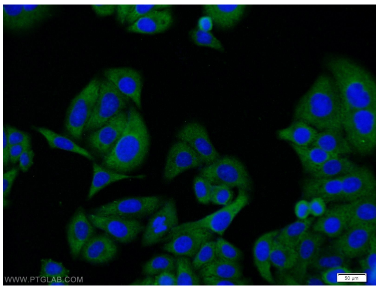

Immunofluorescent analysis of HepG2 cells using 60134-1-Ig (NR1H3 antibody) at dilution of 1:25 and Alexa Fluor 488-conjugated AffiniPure Goat Anti-Mouse IgG (H+L).

PBR/TSPO Rabbit mAb

Catalog No.: A4881 Recombinant 1 Publications

Basic Information

Observed MW
18kDa

Calculated MW
19kDa

Category
Primary antibody

Applications
WB,IHC-P,IF/ICC

Cross-Reactivity
Human, Mouse

CloneNo number
ARC0308

Background

Present mainly in the mitochondrial compartment of peripheral tissues, the protein encoded by this gene interacts with some benzodiazepines and has different affinities than its endogenous counterpart. The protein is a key factor in the flow of cholesterol into mitochondria to permit the initiation of steroid hormone synthesis. Alternatively spliced transcript variants have been reported; one of the variants lacks an internal exon and is considered non-coding, and the other variants encode the same protein.

Recommended Dilutions

**WB**	1:500 - 1:1000
**IHC-P**	1:50 - 1:200
**IF/ICC**	1:50 - 1:200

Immunogen Information

Gene ID
706

Swiss Prot
P30536

Immunogen
A synthetic peptide corresponding to a sequence within amino acids 70-169 of human PBR/TSPO (P30536).

Synonyms
DBI; IBP; MBR; PBR; PBS; BPBS; BZRP; PKBS; PTBR; mDRC; pk18; TSPO1

Contact

www.abclonal.com

Product Information

Source
Rabbit

Isotype
IgG

Purification
Affinity purification

Storage
Store at -20℃. Avoid freeze / thaw cycles.
Buffer: PBS with 0.02% sodium azide,0.05% BSA,50% glycerol,pH7.3.

Validation Data

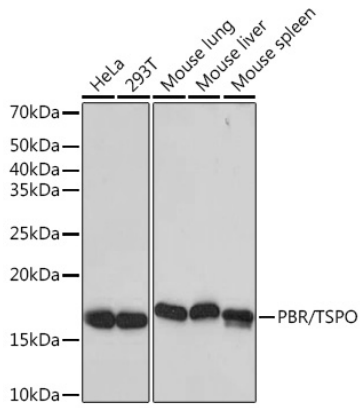

Western blot analysis of extracts from various cell lines, using PBR/TSPO Rabbit mAb (A4881) at 1:1000 dilution.
Secondary antibody: HRP Goat Anti-Rabbit IgG (H+L) (AS014) at 1:10000 dilution.
Lysates/proteins: 25μg per lane.
Blocking buffer: 3% nonfat dry milk in TBST.
Detection: ECL Basic Kit (RM00020).
Exposure time: 1s.

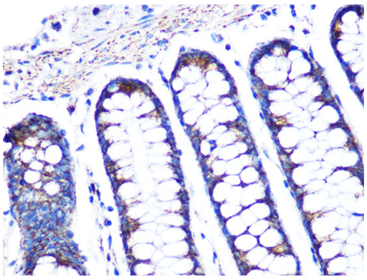

Immunohistochemistry analysis of paraffin-embedded human colon using PBR/TSPORabbit mAb (A4881) at dilution of 1:100 (40x lens).Perform microwave antigen retrieval with 10 mM PBS buffer pH 7.2 before commencing with IHC staining protocol.

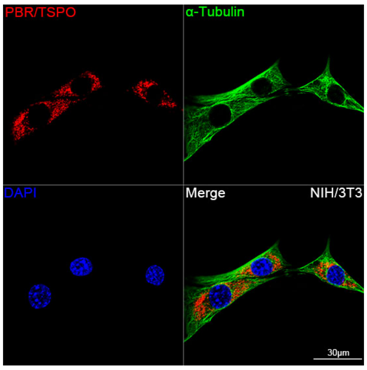

Confocal imaging of NIH/3T3 cells using PBR/TSPO Rabbit mAb (A4881,dilution 1:100)(Red). The cells were counterstained with α-Tubulin Mouse mAb (AC012,dilution 1:400) (Green). DAPI was used for nuclear staining (blue). Objective: 100x.

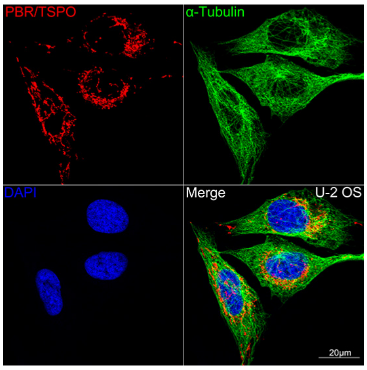

Confocal imaging of U-2 OS cells using PBR/TSPO Rabbit mAb (A4881,dilution 1:100)(Red). The cells were counterstained with α-Tubulin Mouse mAb (AC012,dilution 1:400) (Green). DAPI was used for nuclear staining (blue). Objective: 100x.

Antibody | Protein | ELISA Kits | Enzyme | NGS | Service

For research use only. Not for therapeutic or diagnostic purposes.
Please visit http://abclonal.com for a complete listing of recommended products.
